# Supplementary material for: An analog to digital converter controls bistable transfer competence development of a widespread bacterial integrative and conjugative element
Source: eLife. 2020 Jul 28;9:e57915. doi: 10.7554/eLife.57915 (PMC7423338; doi:10.7554/eLife.57915)
Supplement: Supplementary file 1. [file elife-57915-supp1.docx]

**Table S1 Plasmid and strain numbers of used** *P. putida* UWC1 **derivatives**.

| **Plasmid** | **Description** |
| --- | --- |
| pME6032 | pVS1-p15A shuttle vector carrying the *lacI^q^-P_tac_* expression system, Tc^R^ |
| pME*tciR* | pME6032 derivative allowing IPTG-controlled expression of *tciR* |
| pME*bisR* | pME6032 derivative allowing IPTG-controlled expression of *bisR* |
| pME*bisC* | pME6032 derivative allowing IPTG-controlled expression of *bisC* (old:pME*97*) |
| pME*bisD* | pME6032 derivative allowing IPTG-controlled expression of *bisD* (old:pME*parB*) |
| pME*bisDC* | pME6032 derivative allowing IPTG-controlled expression of *bisCD* (old: pME*parB97*) |
| pME*parA* | pME6032 derivative allowing IPTG-controlled expression of *parA* |
| pME*parAsB* | pME6032 derivative allowing IPTG-controlled expression of *parA, shi* and *bisD* |
| pME*bisC97* | pME6032 derivative allowing IPTG-controlled expression of *bisC* and *96323* (old:pME*9697*) |
| pME*alpA* | pME6032 derivative allowing IPTG-controlled expression of *alpA* |
| pME*inrR* | pME6032 derivative allowing IPTG-controlled expression of *inrR* |
| pME*reg* | pME6032 derivative allowing IPTG-controlled expression of the *alpA-inrR* locus (old: pMEEE6.4*alpA*) |
| pME*reg*∆alpA | pME6032 derivative allowing IPTG-controlled expression of the *parA-inrR* locus (old:pME EE6.4) |
| pME*reg*∆alpA∆P | pMEEE6.4 derivative lacking 3’ half of *96323*, *952313* and *inrR* (old: pMEEE6.4PstI) |
| pME*reg*∆alpA∆A | pMEEE6.4 derivative lacking the *bisC*, *96323*, *95213* and *inrR* (old: pMEEE6.4AfeI) |
| pME*reg*∆P | pMEEE6.4*alpA* derivative lacking 3’ half of *96323*, *952313* and *inrR* (old: pMEEE6.4alpA∆PstI) |
| pME*reg*∆A | pMEEE6.4*alpA* derivative lacking the *bisC*, *96323*, *95213* and *inrR* (old: pMEEE6.4alpA∆AfeI) |
| pME*bg* (component B) | *lacI^q^-P_tac_*-less pME6032 derivative carrying the *P_alpA_-inrR* locus |
| pME*bg_short* (compB’) | pME*bg* derivative lacking 3’ half of *96323*, *952313* and *inrR* |

| **Strain number** | **Strain description** |
| --- | --- |
| **2737** | ***P. putida* UWC1 ICE*clc*** inserted at *glytRNA5*gene |
| 6144 | Strain 2737 with pME6032 |
| 6316 | Strain 2737 with pME*tciR* |
| 6317 | Strain 2737 with pME*bisR* |
| 6019 | Strain 2737 with pME*reg*∆A |
| 4479**-**4480-4481 | ***P. putida* ICE*clc*∆*tciR* with miniTn5::*P_inR_-echerry/P_int_-egfp***, Km^R^ |
| 5827-5828-5829 | Strain 4479, 4480 or 4481 with pME*bisR* |
| 6092-6093-6094 | Strain 4479, 4480 or 4481 with pME*bisCD* |
| 5563**-**5564-5565 | ***P. putida* ICE*clc*∆*bisR with* miniTn5::*P_int_-echerry/P_inR_-egfp***, Km^R^ |
| 6321-6322-6323 | Strain 5563, 5564 or 5565 with pME6032 |
| 6324-6325-6326 | Strain 5563, 5564 or 5565 with pME*tciR* |
| 5805-5806-5807 | Strain 5563, 5564 or 5565 with pME*bisR* |
| 6095-6096-6097 | Strain 5563, 5564 or 5565 with pME*bisDC* |
| 6168-6170-6172 | ***P. putida* ICE*clc*∆*bisD* with miniTn5::*P_int_-echerry/P_inR_-egfp***, Km^R^ |
| 6327-6331-6335 | Strain 6168, 6170 or 6172 with pME6032 |
| 6328-6332-6336 | Strain 6168, 6170 or 6172 with pME*tciR* |
| 6328-6333-6337 | Strain 6168, 6170 or 6172 with pME*bisR* |
| 6330-6334-6338 | Strain 6168, 6170 or 6172 with pME*bisDC* |
| 4344 | ***P. putida* UWC1 miniTn7::*P_inR_-egfp***, Gm^R^ |
| 5502 | Strain 4344 with pME6032 |
| 5586 | Strain 4344 with pME*tciR* |
| 4510 | Strain 4344 with pME*reg* |
| 6856 | Strain 4344 with pME*bisDC* |
| 5690-5691-5692 | ***P. putida* UWC1 miniTn5::*P_int_-echerry/P_inR_-egfp***, Km^R^ |
| 5719-5725-5731 | Strain 5690, 5691 or 5692 with pME6032 |
| 5853-5854-5855 | Strain 5690, 5691 or 5692 with pME*bisR* |
| 6059-6060-6061 | Strain 5690, 5691 or 5692 with pME*bisDC* |
| 5884-5887-5890 | Strain 5690, 5691 or 5692 with pME*parB* |
| 5724-5730-5736 | Strain 5690, 5691 or 5692 with pME*bisC* |
| 5721-5727-5733 | Strain 5690, 5691 or 5692 with pME*reg* |
| 5720-5726-5732 | Strain 5690, 5691 or 5692 with pME*reg∆alpA* |
| 6045-6049-6053 | Strain 5690, 5691 or 5692 with pME*reg∆P* |
| 6043-6047-6051 | Strain 5690, 5691 or 5692 with pME*reg∆alpA∆P* |
| 6042-6046-6050 | Strain 5690, 5691 or 5692 with pME*reg∆alpA∆A* |
| 5871-5872-5873 | Strain 5690, 5691 or 5692 with miniTn*7*::P_tac_-*bisR* |
| 5985-5986-5987 | Strain 5690, 5691 or 5692 with pME*bg* |
| 5916-5917-5918 | Strain 5871, 5872 or 5872 with pME*bg* |
| 5978-5981-5984 | Strain 5871, 5872 or 5872 with pME*bg-short* |
| 6376 | ***P. putida* strain 5690 with inserted ICE*clc***, Km^R^ |
| 6423 | Strain 6376 with pME6032 |
| 6424 | Strain 6376 with pME*tciR* |
| 6425 | Strain 6376 with pME*bisR* |
| 6427 | Strain 6376 with pME*bisC* |
| 6426 | Strain 6376 with pME*bisD* |
| 6428 | Strain 6376 with pME*bisDC* |
| 4347 | ***P. putida* UWC1 miniTn7::*P_alpA_-egfp***, Gm^R^ |
| 5503 | Strain 4347 with pME6032 |
| 5587 | Strain 4347 with pME*tciR* |
| 5561 | Strain 4347 with pME*bisR* |
| 5517 | Strain 4347 with pME*alpA* |
| 5533 | Strain 4347 with pME*bisC96* |
| 5821 | Strain 4347 with pME*inrR* |
| 5892 | Strain 4347 with pME*parA* |
| 4489 | Strain 4347 with pME*parAsB* |
| 5893 | Strain 4347 with pME*bisD* |
| 6080 | Strain 4347 with pME*bisC* |
| 6065 | Strain 4347 with pME*bisDC* |
| 6081 | Strain 4347 with pME*reg*∆alpA∆P |
| 5688, 5689 | ***P. putida* UWC1 miniTn5::*P_bisR_-egfp***, Km^R^ |
| 5698-5699 | Strain 5688 or 5689 with pME6032 |
| 5700-5701 | Strain 5688 or 5689 with pME*tciR* |
| 5991 | *P. putida* UWC1 miniTn7::*P_tac_-echerry*, Gm^R^ |
