# Supplementary material for: An analog to digital converter controls bistable transfer competence development of a widespread bacterial integrative and conjugative element
Source: eLife. 2020 Jul 28;9:e57915. doi: 10.7554/eLife.57915 (PMC7423338; doi:10.7554/eLife.57915)
Supplement: Supplementary file 2. [file elife-57915-supp2.docx]

*Pseudomonas putida* type 21 C defined minimal medium (Gerhard *et al*., 1981):

(per liter)

NH_4_Cl 1.00 g

Na_2_HPO_4_ . 2H_2_O 3.49 g

KH_2_PO_4_ 2.77 g

pH 6.8, sterilize by autoclaving afterwards add:

Hutner’s vitamin free mineral base (sterile) 20 ml

vitamins (500 x) sterile 2 ml

Hutner’s vitamin free mineral base:

(per liter)

NTA (nitrilotriacetic acid, chelating agent) 10 g

MgSO_4_ . 7 H_2_O 14.45 g

CaCl_2_ . 2H_2_O 3.33 g

(NH_4_)_6_Mo_7_O_24_ . 4 H_2_O 9.74 mg

FeSO_4_ . 7 H_2_O 99 mg

Metals 44 50 ml

Add each compound in this order. Wait until compound is entirely dissolved prior to adding the next one.

sterilize by filtration and store it at 4 °C.

Metals 44:

(per 100 ml)

Na_4_EDTA . 4 H_2_O 387 mg

ZnSO_4_ . 7 H_2_O 1.095 g

FeSO4 . 7H_2_O 914 mg

MnSO_4_. H_2_O 154 mg

CuSO_4_ . 5 H_2_O 39.2 mg

Co(NO_3_)_2_ . 6 H_2_O 24.8 mg

Na_2_B_4_O_7_.10 H_2_O 17.7 mg

+ a few trops of 6 N H_2_SO_4_

sterilize by filtration and store it at 4 °C.

Vitamin solution (500 x):

(per 100 ml)

Biotin 0.5 mg

Nicotinic acid 50 mg

thiamin hydrochloride 25 mg

sterilize by filtration and store it at 4 °C.
