# Supplementary material for: An analog to digital converter controls bistable transfer competence development of a widespread bacterial integrative and conjugative element
Source: eLife. 2020 Jul 28;9:e57915. doi: 10.7554/eLife.57915 (PMC7423338; doi:10.7554/eLife.57915)
Supplement: Supplementary file 3. [file elife-57915-supp3.docx]

Table. Primers used in this study

| **Name** | **Nucleotide sequence (5’ to 3’)^a^** |  |
| --- | --- | --- |
| tciREcorI.F | GGGGGGGAATTCGCTGAACGTCCGTGGTT | Amplification of *tciR* |
| tciREcorI.R | GGGGGGGAATTCCAGAACCACGGCCGTGAA |  |
| 101284EcoRI.F | GGGGGGGAATTCCCGCAGATTCGGGCTTGCAA | Amplification of *bisR* |
| 101284EcoRI.R | GGGGGGGAATTCGGAGTCGGCCATGTAGTTCTT |  |
| 97571EcoRI.F | GGGGGGGAATTCTTATCAACCCAATCCTTGATCGAT | Amplification of *bisC* |
| 97571RBSEcoRI.R | TTTTTTGAATTCGGAAGCCGGCGCTGTCGGTT |  |
| parBEcoRI.F | GGGGGGGAATTCCCGAGCGCCAGGCCCAA | Amplification of *bisD* |
| 96323EcoRI.F | TTTTTTGAATTCCGATCAGCTCTTGCGCAGAA |  |
| KpnI_98147_For | TTTTGGTACCCTTGATTTCCTCGTAGGCGG | Addition of *alpA* |
| EcoRI_100952_Rev2 | TTTTGAATTCACTGAGCGTTCAGGAGTG |  |
| Fw_101284_(BamHI) | TTTTTGGATCCATGCTCCGTCTCCTTCCAGGA | Amplification of *bisR* promoter |
| Rev_101284_(XbaI) | TTTTTTCTAGACGCAGTCGTCACAACGTCAT |  |
| Fw_100952_(BamHI) | TTTTTGGATCCTTCATCGAGACGCAAGATGC | Amplification of *alpA* promoter |
| Rev_100952_(XbaI) | TTTTTTCTAGAATTACCGATCGCACGCTGCAA |  |
| up-101284-F-XmaI | TTTTCCCGGGGGTCGTTGGAGAGCACTAG | Upstream fragment for *bisR* deletion |
| Up-101284-R | CTCATGGGCATTACCGATC |  |
| dn-101284-rc-up | GATCGGTAATGCCCATGAGGGTGCATCATTCTTGTTCTC | Downstream fragment for *bisR* deletion |
| Dn-101284-R-XbaI | TTTTTCTAGACCAACAAAGATCGTCCAAAG |  |
| del3 for1 Bam | TTTTTGGATCC GTCGTGCAGAACATAGAGGTT | Upstream fragment for *bisD* deletion |
| del3 rev1 Hind | TTTTTACTAGT ACGAGGGTTGCCAGCATGTCC |  |
| del3 for2 Hind | TTTTTACTAGT TCAGCCATGGCCGCATTCCTT | Downstream fragment for *bisD* deletion |
| Del3 rev2 Pst | TTTTTAAGCTT GCGGCTCTCCCTCGGAATTCC |  |
| eCherrySpeI.F | AACAGAATTCGAGCTCCTCAACTAGTAGAAGGAGCACCTGCCATGGT | Recloning of *echerry* with SpeI sites |
| eCherrySpeI.R | CCTGCAGCCCGGGGGATCCAACTAGTTTATTTGTACAGCTCATCCATGCCA |  |

**^a^** restriction sites are underlined
