# Supplementary material for: An analog to digital converter controls bistable transfer competence development of a widespread bacterial integrative and conjugative element
Source: eLife. 2020 Jul 28;9:e57915. doi: 10.7554/eLife.57915 (PMC7423338; doi:10.7554/eLife.57915)
Supplement: Supplementary file 4. [file elife-57915-supp4.pdf]

SUPPLEMENTARY MATERIAL
ICEclc transfer competence bistable regulatory model

REACTIONS

A: BisDC feedback loop on alpA-promoter
B: BisDC feedback loop on alpA-promoter, initiated by BisR
C: TciR activating bisR-promoter
D: BisDC activating three downstream (late) promoters
E: BisDC activating GFP reporter.

$$\begin{aligned}
2\mathrm{BisCD} + \mathrm{PalpA}^{OFF} &\underset{\kappa_b^{BisCD}}{\overset{\kappa_f^{BisCD}}{\rightleftharpoons}} \mathrm{PalpA}^{ON}_{BisCD} \\
\mathrm{PalpA}^{ON}_{BisCD} &\xrightarrow{\mu^{BisCD}} \mathrm{PalpA}^{ON}_{BisCD} + \mathrm{BisCD} \\
\mathrm{BisCD} &\xrightarrow{\nu^{BisCD}} \emptyset
\end{aligned} \tag{A}$$

$$\begin{aligned}
4\mathrm{BisR} + \mathrm{PalpA}^{OFF} &\underset{\kappa_b^{BisR}}{\overset{\kappa_f^{BisR}}{\rightleftharpoons}} \mathrm{PalpA}^{ON}_{BisR} \\
\mathrm{PalpA}^{ON}_{BisR} &\xrightarrow{\mu^{BisR}} \mathrm{PalpA}^{ON}_{BisR} + \mathrm{BisCD} \\
\emptyset &\xrightarrow{\mu_0^{BisR}} \mathrm{BisR} \\
\mathrm{BisR} &\xrightarrow{\nu^{BisR}} \emptyset
\end{aligned} \tag{B}$$

$$\begin{aligned}
\emptyset &\underset{\nu^{TciR}}{\overset{\mu_0^{TciR}}{\rightleftharpoons}} \mathrm{TciR} \\
4\mathrm{TciR} + \mathrm{P}\mathit{bisR}^{OFF} &\xrightarrow{\kappa_f^{TciR}} \mathrm{PbisR}^{ON} \\
\mathrm{P}\mathit{bisR}^{ON} &\xrightarrow{\kappa_b^{TciR}} 4\mathrm{TciR} + \mathrm{P}\mathit{bisR}^{OFF} \\
\mathrm{P}\mathit{bisR}^{ON} &\xrightarrow{\mu_1^{TciR}} \mathrm{P}\mathit{bisR}^{ON} + 10\mathrm{BisR}
\end{aligned} \tag{C}$$

$$\begin{aligned}
2\mathrm{BisCD} + \mathrm{P}\mathit{int}^{OFF} &\underset{\kappa_b^{int}}{\overset{\kappa_f^{int}}{\rightleftharpoons}} \mathrm{P}\mathit{int}^{ON} \\
2\mathrm{BisCD} + \mathrm{P}\mathit{late2}^{OFF} &\underset{\kappa_b^{late2}}{\overset{\kappa_f^{late2}}{\rightleftharpoons}} \mathrm{P}\mathit{late2}^{ON} \\
2\mathrm{BisCD} + \mathrm{P}\mathit{late3}^{OFF} &\underset{\kappa_b^{late3}}{\overset{\kappa_f^{late3}}{\rightleftharpoons}} \mathrm{P}\mathit{late3}^{ON}
\end{aligned} \tag{D}$$

$$\begin{aligned}
2\mathrm{BisCD} + \mathrm{P}\mathit{GFP}^{OFF} &\underset{\kappa_b^{GFP}}{\overset{\kappa_f^{GFP}}{\rightleftharpoons}} \mathrm{P}\mathit{GFP}^{ON} \\
\mathrm{P}\mathit{GFP}^{ON} &\xrightarrow{\mu^{GFP}} \mathrm{P}\mathit{GFP}^{ON} + \mathrm{GFP} \\
\mathrm{GFP} &\xrightarrow{\nu^{GFP}} \emptyset
\end{aligned} \tag{E}$$

(1)

List of variables	
Variable	Explanation
BisCD	Activator protein complex for P_{alpA} and for late promoters
P_{alpA}	Promoter driving expression of *bisCD*
BisR	Activator protein for P_{alpA}
TciR	Activator protein for P_{bisR}
P_{bisR}	Promoter driving expression of *bisR*
P_{int}	(Late) promoter driving expression of the integrase
P_{late2}	Late promoter nr 2 activated by BisCD
P_{late3}	Late promoter nr 3 activated by BisCD
P_{GFP}	Late promoter controlling artificial expression of GFP, activated by BisCD

List of parameters			
Parameter	Value	Symbol in Julia	Explanation
κ_f^{BisCD}	1.0 or 5.0	A1	binding rate of BisCD to P_{alpA}
κ_b^{BisCD}	1.0 or 5.0	A2	unbinding rate of BisCD to P_{alpA}
μ^{BisCD}	1.0	A3	production rate of BisCD when BisCD is bound to P_{alpA}
ν^{BisCD}	0.17 - 0.3	A4	degradation rate of BisCD
κ_f^{BisR}	1.0	B1	binding rate of BisR to P_{alpA}
κ_b^{BisR}	1.0	B2	unbinding rate of BisR to P_{alpA}
μ^{BisR}	1.0	B3	production rate of BisCD when BisR is bound to P_{alpA}
μ_0^{BisR}	0.0 - 1.0	B4	basal production rate of BisR
ν^{BisR}	0.17	B5	degradation rate of BisR
μ_0^{TciR}	0.35	C1	basal production rate of TciR
ν^{TciR}	0.17	C2	degradation rate of TciR
κ_f^{TciR}	1.0	C3	binding rate of TciR to P_{bisR}
κ_b^{TciR}	1.0	C4	unbinding rate of TciR to P_{bisR}
μ_1^{TciR}	1.0	C5	production rate of BisR when TciR is bound to P_{bisR}
κ_f^{int}	1.0	D1	binding rate of BisCD to P_{int}
κ_b^{int}	1.0	D2	unbinding rate of BisCD to P_{int}
κ_f^{late2}	1.0	D3	binding rate of BisCD to P_{late2}
κ_b^{late2}	1.0	D4	unbinding rate of BisCD to P_{late2}
κ_f^{late3}	1.0	D5	binding rate of BisCD to P_{late3}
κ_b^{late3}	1.0	D6	unbinding rate of BisCD to P_{late3}
κ_f^{GFP}	1.0	E1	binding rate of BisCD to P_{GFP}
κ_b^{GFP}	1.0	E2	unbinding rate of BisCD to P_{GFP}
μ^{GFP}	1.0	E3	production rate of GFP when BisCD is bound to P_{GFP}
ν^{GFP}	0 or 0.17	E4	degradation rate of GFP
