# Supplementary material for: An analog to digital converter controls bistable transfer competence development of a widespread bacterial integrative and conjugative element
Source: eLife. 2020 Jul 28;9:e57915. doi: 10.7554/eLife.57915 (PMC7423338; doi:10.7554/eLife.57915)
Supplement: Transparent reporting form [file elife-57915-transrepform.docx]

***eLife’s* transparent reporting form**

We encourage authors to provide detailed information *within their submission* to facilitate the interpretation and replication of experiments. Authors can upload supporting documentation to indicate the use of appropriate reporting guidelines for health-related research (see [EQUATOR Network](http://www.equator-network.org/%20)), life science research (see the [BioSharing Information Resource](https://biosharing.org/)), or the [ARRIVE guidelines](http://www.plosbiology.org/article/info:doi/10.1371/journal.pbio.1000412) for reporting work involving animal research. Where applicable, authors should refer to any relevant reporting standards documents in this form.

If you have any questions, please consult our Journal Policies and/or contact us: [editorial@elifesciences.org](mailto:editorial@elifesciences.org).

**Sample-size estimation**

- You should state whether an appropriate sample size was computed when the study was being designed
- You should state the statistical method of sample size computation and any required assumptions
- If no explicit power analysis was used, you should describe how you decided what sample (replicate) size (number) to use

Please outline where this information can be found within the submission (e.g., sections or figure legends), or explain why this information doesn’t apply to your submission:

Central to the study is the determination of subpopulation sizes expressing key promoters of the ICE, which are representative for ICE bistability behaviour. Subpopulations are determined from quantile-quantile plotting of fluorescence values of eGFP or eCherry expressed from transcriptional fusions to one of the ICE bistably expressed promoters inserted in single copy in the bacterial chromosome (Reinhard F, van der Meer JR (2013). Improved statistical analysis of low abundance phenomena in bimodal bacterial populations. *PLoS ONE* **8:** e78288.) Two subpopulations (a ‘main’ and a ‘small’) will produce two intersecting lines in qq-plots. This is explained in l. 558-573 of the main text. Examples where qq-plotting is used indicate the number of cells on which the analysis is based. The typical number of cells was between 500 and 1500, derived from 6-12 images of the same culture (technical replicates).

**Replicates**

- You should report how often each experiment was performed
- You should include a definition of biological versus technical replication
- The data obtained should be provided and sufficient information should be provided to indicate the number of independent biological and/or technical replicates
- If you encountered any outliers, you should describe how these were handled
- Criteria for exclusion/inclusion of data should be clearly stated
- High-throughput sequence data should be uploaded before submission, with a private link for reviewers provided (these are available from both GEO and ArrayExpress)

Please outline where this information can be found within the submission (e.g., sections or figure legends), or explain why this information doesn’t apply to your submission:

Analysis of ICE promoter activation in *P. putida* with or without ICE and its deletion variants is based on calculation of the mean of the 75^th^ percentile or the median fluorescence intensity among single cells in biological triplicate cultures. Every individual replicate contained between 500-1500 cells, imaged and summed from 6-12 positions (technical replicates). This is described in l. 564-573 of the main manuscript and further specified in each relevant figure legend. Induction was judged from comparison to the fluorescence of a control strain carrying an empty plasmid using a pair-wise t-test, or, in case of a coherent set of strains measured simultaneously, by ANOVA followed by a post-hoc tukey test. This is specified in each relevant figure legend.

Source data are provided with the raw data for each of the figure displays. In a few cases of strains with negative responses, only a single individual replicate was used, in which case the technical replicates were used to calculate the mean of the median fluorescence intensity. This is specified in the raw source data.

Analysis of the ICE transfer frequencies is based on colony forming units of exconjugants on selective plates compared to that of donors in the same assay. This was calculated from independent biological replicate filter matings, as descibed in l. 526-533 of the main manuscript and further in relevant parts of the figure legends where such data are presented. Statistical significance was tested in ANOVA followed by post-hox Tukey test, as mentioned in l. 572-575. Raw source data of all mating experiments are provided.

**Statistical reporting**

- Statistical analysis methods should be described and justified
- Raw data should be presented in figures whenever informative to do so (typically when N per group is less than 10)
- For each experiment, you should identify the statistical tests used, exact values of N, definitions of center, methods of multiple test correction, and dispersion and precision measures (e.g., mean, median, SD, SEM, confidence intervals; and, for the major substantive results, a measure of effect size (e.g., Pearson's r, Cohen's d)
- Report exact p-values wherever possible alongside the summary statistics and 95% confidence intervals. These should be reported for all key questions and not only when the p-value is less than 0.05.

Please outline where this information can be found within the submission (e.g., sections or figure legends), or explain why this information doesn’t apply to your submission:

Statistical procedures are described in l. 566-575 of the main text. Raw cell numbers as well as the number of replicates are reported in the figure legends. Standard deviations from the means are indicated as well as results of the statistical tests.

Source files are included for every figure display.

(For large datasets, or papers with a very large number of statistical tests, you may upload a single table file with tests, Ns, etc., with reference to sections in the manuscript.)

**Group allocation**

- Indicate how samples were allocated into experimental groups (in the case of clinical studies, please specify allocation to treatment method); if randomization was used, please also state if restricted randomization was applied
- Indicate if masking was used during group allocation, data collection and/or data analysis

Please outline where this information can be found within the submission (e.g., sections or figure legends), or explain why this information doesn’t apply to your submission:

Not relevant

**Additional data files (“source data”)**

- We encourage you to upload relevant additional data files, such as numerical data that are represented as a graph in a figure, or as a summary table
- Where provided, these should be in the most useful format, and they can be uploaded as “Source data” files linked to a main figure or table
- Include model definition files including the full list of parameters used
- Include code used for data analysis (e.g., R, MatLab)
- Avoid stating that data files are “available upon request”

Please indicate the figures or tables for which source data files have been provided:

Source data have been provided for all figure display elements in a single ZIP. This contains subfolders labeled e.g., Fig2a-source-data.
